# The impact of time between surgery and adjuvant chemoradiotherapy in advanced oral cavity squamous cell carcinoma

**DOI:** 10.3389/fonc.2024.1393910

**Published:** 2024-05-07

**Authors:** Friedrich Mrosk, Majd Absah, Maximilian Richter, Erin Sprünken, Christian Doll, Kilian Kreutzer, Carsten Rendenbach, Marcus Beck, Konrad Klinghammer, Max Heiland, Steffen Koerdt

**Affiliations:** ^1^ Department of Oral and Maxillofacial Surgery, Charité – Universitätsmedizin Berlin, Corporate Member of Freie Universität Berlin and Humboldt-Universität zu Berlin, Berlin, Germany; ^2^ Institute of Biometry and Clinical Epidemiology, Charité – Universitätsmedizin Berlin, Corporate Member of Freie Universität Berlin and Humboldt-Universität zu Berlin, Berlin, Germany; ^3^ Department of Radiation Oncology, Charité – Universitätsmedizin Berlin, Corporate Member of Freie Universität Berlin and Humboldt-Universität zu Berlin, Berlin, Germany; ^4^ Department of Hematology, Oncology and Cancer Immunology, Corporate Member of Freie Universität Berlin and Humboldt-Universität zu Berlin, Berlin, Germany

**Keywords:** oral squamos cell carcinoma, oncological prognosis, adjuvant treatment, delay, chemoradiation

## Abstract

**Objective:**

In advanced oral squamous cell carcinoma (OSCC), adjuvant therapy (AT) is an important part of the treatment to ensure extended locoregional control after primary surgical resection. The impact of the time interval between surgery and AT on the oncological prognosis remains unclear, particularly in high-risk constellations. The aim of this study is to categorize treatment delays and to determine their impact on the oncological prognosis within the context of the histopathological risk parameters of patients with advanced OSCC.

**Methods:**

In this single-institutional retrospective cohort study, all patients treated for OSCC between 2016 and 2021 and who received postoperative chemoradiation (POCRT) were included. Patients were divided into two groups: Group I: ≤ 6 weeks between surgery and POCRT; and Group II: > 6 weeks between surgery and POCRT.

**Results:**

Overall, 202 patients were included (Group I: 156 (77.2%) vs. Group II: 46 (22.8%)). There were no statistically significant differences in epidemiological aspects and histopathological risk factors between the two groups. The maximum time to initiation of POCRT was 11 weeks. Delayed POCRT initiation had no statistically significant influence on the 5-year OS (61.6% vs. 57.3%, p = 0.89), locoregional control rate (38.6% vs. 43.3%, p = 0.57), and RFS (32.3% vs. 30.4%, p = 0.21). On multivariate analysis, extracapsular spread (HR: 2.21, 95% CI: 1.21 – 4.04, p = 0.01) and incomplete surgical resection (HR: 2.01, 95% CI: 1.10 – 3.69, p = 0.02) were significantly correlated with OS. For RFS, ECS (HR: 1.82, 95% CI: 1.15 – 2.86, p = 0.01), incomplete resection (HR: 1.67, 95% CI: 1.04 – 2.71, p = 0.04), and vascular infiltration of the tumor (V-stage; HR: 2.15, 95% CI: 1.08 – 4.27, p = 0.03) were significant risk predictors.

**Conclusion:**

Delays in POCRT initiation up to 11 weeks after surgical resection for advanced OSCC were not statistically significantly associated with impaired survival. In cases of prolonged surgical treatment due to management of complications, a small delay in AT beyond the recommended time limit may be justified and AT should still be pursued.

## Introduction

Depending on the constellation of the histopathological risk factors, adjuvant therapy (AT) is an important part in the treatment of oral cavity squamous cell carcinoma (OSCC) to ensure extended locoregional control after surgical resection, especially in advanced stage diseases. According to international guidelines, adverse risk factors that favor postoperative radiation therapy (PORT) include cervical lymph node metastasis (CLNM), extracapsular spread (ECS), close or positive resection margins, advanced T-stages (pT3–4) as well as vascular, perineural, and lymphatic invasion (1, 2). Moreover, studies have shown that patients with higher risk constellations such as ECS and positive margins particularly benefit from radiation with concomitant chemotherapy (POCRT), with significant differences in survival and locoregional relapse of 10–13% (3–5).

The current guideline of the American National Comprehensive Cancer Network (NCCN), as well as most European guidelines, have established 6 weeks as a recommended time frame between surgery and the start of AT (1, 2, 6, 7). In addition, the German national guideline and the European ESMO guideline have also stated that the time between surgery and the end of AT should not exceed 11 weeks (1, 6). Various factors may delay initiation of AT, such as wound healing complications; postoperative infection; patients wish or initial refusal; distance to treatment centers; or reduced compliance (8). In an analysis of the national Cancer Data Base (NCDB), non-adherence to the guideline recommended time limit of 6 weeks was present in 55.7% of the cases (9). These situations give rise to the question of the role of AT beyond the recommended time limit, and if patients still benefit from AT if there is a treatment delay. Several studies have investigated the role of time delays in the treatment process (10–13). However, the results of these studies are diverging, reporting either benefits in survival or no influence regarding recommended time limits. In addition, there is evidence that the oncological outcome as well as response to adjuvant treatment of head and neck squamous cell carcinoma (HNSCC) varies between tumor sites (5, 14, 15). The aim of this study is therefore to determine the time dependence of patients who received POCRT due to higher-risk constellations after primary surgical treatment of advanced OSCC only.

## Methods

### Study design

In this single-institutional retrospective cohort study, all patients who were treated for OSCC by primary surgical resection at Charité – University Medicine Berlin in combination with neck dissection and who received and completed recommended, adjuvant POCRT between 2016 and 2021 were assessed and screened for inclusion. Patients with accompanying secondary carcinoma, distant metastases at the time of primary treatment (UICC stage IVc), or incompletely performed POCRT were excluded. Demographic data included age at the time of surgery, and sex. Assessed histopathological risk factors included the TNM-staging according the 8th AJCC Cancer Staging Manual including depth of invasion (DOI), ECS and the grade of differentiation, presence of lymphatic and vascular infiltration, and surgical margin status (16). Margin status was dichotomized into < 5mm and ≥ 5mm according to the current guidelines (1, 2). The times between surgery and the start of POCRT and the completion of POCRT (treatment package time) were assessed. Patients were divided into two groups according to the initiation time between surgery and POCRT according to the current guideline recommendations:

- Group I: ≤ 6 weeks between surgery and the first day of POCRT.
- Group II: > 6 weeks between surgery and the first day of POCRT (treatment delay).

The time in weeks was rounded as a whole number in each case. Ethical approval was given by the institutional ethics committee (EA2/077/20).

### Statistical analysis

Descriptive data was presented as absolutes as well as means with their standard deviations (± SD). Both study groups were compared using the Chi-square test in terms of the distribution of histopathological risk factors. Overall survival (OS), locoregional control rate (LRCR), and recurrence free survival (RFS) were determined by Kaplan-Meier analysis. Survival curves were statistically analyzed using the log rank test. In addition, univariate and multivariate Cox regression was performed to determine correlations between risk factors and RFS. Hazard ratios (HR) were presented with their respective 95% confidence intervals (CI). Statistically significant parameters were then considered for multivariate analysis. P-values < 0.05 were considered statistically significant. All statistical analyses were conducted using SPSS version 28 (IBM Corp., USA).

## Results

### Patient characteristics

Overall, 202 patients were included into this study. Group I (≤ 6 weeks AT initiation) consisted of 156 patients and 46 patients (22.8%) who experienced a treatment delay beyond the recommended time limit were assigned to Group II. The mean age of the whole study cohort was 62.2 years (± 10.2) and there was no statistically significant difference between both groups (p = 0.43). Furthermore, 123 (60.9%) patients were male and 79 (39.1%) were female, with no significant differences between both groups (p = 1). The most common concurrent chemotherapy regimen was cisplatin in 77.5% of the cases with overall dosage of 200mg/m^2^ weekly administered. In 15.8% of the cases, cisplatin was combined with 5-fluorouracil or mitomycin. In the remaining 6.7% of the cases, cetuximab was administered.

### Time intervals contributing to treatment delay

The mean duration in days of postoperative intensive care unit stay was 1.7 (± 0.6). There was a statistically significant difference between both groups (1.8 ± 0.6 versus 1.6 ± 0.5, p = 0.02). In addition, the mean duration in days of overall hospital stay in the surgical department was 13.5 (± 1.7). There was no statistically significant difference between both groups (13.7 ± 1.7 versus 13.1 ± 1.7, p = 0.06).

The mean duration between surgery and completion of histopathological analysis was 11.8 (± 8.1) days in Group I versus 10.5 (± 4.1) days in Group II (p = 0.51). Furthermore, the mean duration between surgery and recommendation for adjuvant treatment was 16.6 (± 8.7) days in Group I versus 23.1 (± 10.4) days in Group II (p < 0.01). The mean duration between recommendation for adjuvant treatment and the start of AT was 22.3 (± 9.9) days in Group I versus 38.8 (± 14.9) days in Group II (p < 0.01).

### Histopathological risk factors

Histopathological risk factors are shown in **Table 1**. Overall, there were no statistically significant differences in risk factors between both study groups.

**Table 1:** Histopathological risk factors between both study groups after adjustment for confounding bias.

	All (n = 202)	Group I (n = 156)	Group II (n = 46)	p-Value
Disease stage				0.09
pT1-2	85 (42.1)	71 (45.5)	14 (30.4)	
pT3-4	117 (57.9)	85 (55.5)	32 (69.6)	
Nodal status				0.10
pN0	43 (21.3)	29 (18.6)	14 (30.4)	
pN+	159 (78.7)	127 (81.4)	32 (69.6)	
UICC-stage				0.21
III	42 (20.8)	36 (23.1)	6 (13.0)	
IVa	119 (58.9)	87 (55.8)	32 (69.6)	
IVb	41 (20.3)	33 (21.2)	8 (17.4)	
Grade of differentiation				0.49
Grade 1	7 (3.5)	4 (2.6)	3 (6.5)	
Grade 2	148 (73.3)	114 (73.1)	34 (73.9)	
Grade 3	45 (22.3)	36 (23.1)	9 (19.6)	
Grade 4	2 (1.0)	2 (1.3)	0 (0)	
R-stage				0.82
R0	171 (84.7)	131 (84.0)	40 (87.0)	
R1	31 (15.3)	25 (16.0)	6 (13.0)	
Lymphatic invasion				1
Yes	42 (20.8)	33 (21.2)	9 (19.6)	
No	160 (79.2)	123 (78.8)	37 (80.4)	
Vascular invasion				1
Yes	10 (5.0)	8 (5.1)	2 (4.3)	
No	192 (95.0)	148 (94.9)	44 (95.7)	
Close margins				0.38
< 5mm	133 (65.8)	100 (64.1)	33 (71.7)	
≥ 5mm	69 (34.2)	56 (35.9)	13 (28.3)	
Extracapsular spread				0.68
Yes	41 (20.3)	33 (21.2)	8 (23.9)	
No	161 (79.7)	123 (78.8)	38 (76.1)	
Depth of invasion (mean, ± SD)	6.7 (4.1)	6.4 (4.0)	9.0 (4.2)	0.12

### The impact of time between surgery and POCRT initiation

The mean time between surgery and POCRT initiation was 5.5 weeks (± 1.7), ranging between 4 and 11 weeks. In cases of initiation delay (Group II), the mean time between surgery and the start of POCRT was 8.1 weeks (± 1.3). The mean treatment package time was 11.7 weeks (± 3.0), ranging between 8 and 20 weeks.

**Figure 1:**
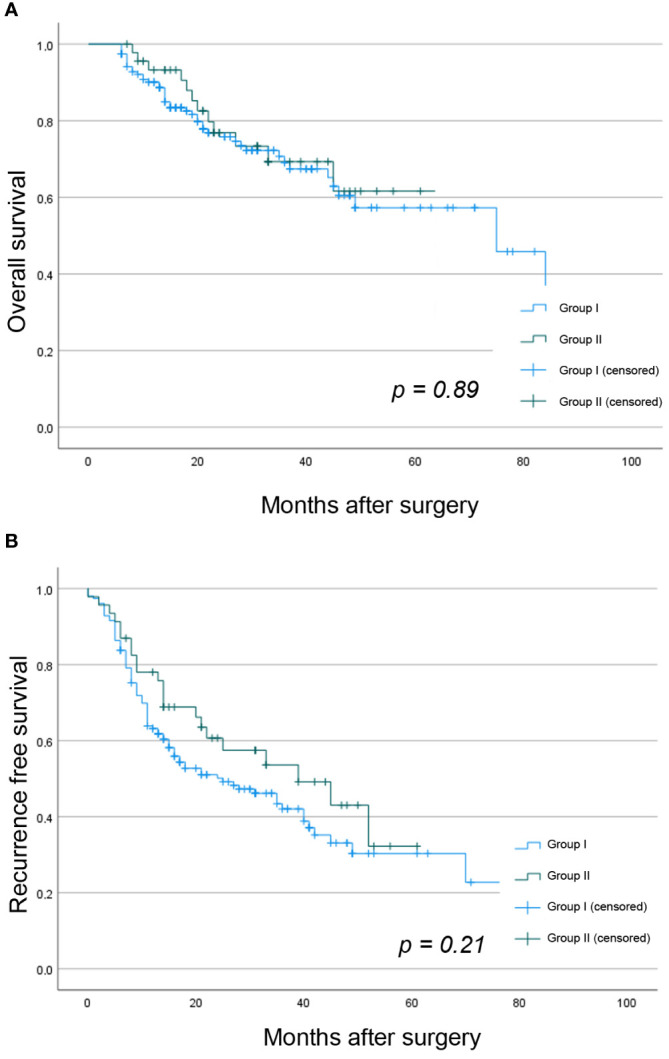
Kaplan Meier analysis showing the difference between both study groups in **(A)** OS and **(B)** RFS.

Overall 5-year OS, LRCR, and RFS for the whole study cohort was 58.2%, 41.6%, and 30.6%, respectively. There were no statistically significant differences between both groups in 5-year OS (61.6% vs. 57.3%, p = 0.89), 3-year LCRC (38.6% vs. 43.3%, p = 0.57), or 3-year RFS (32.3% vs. 30.4%, p = 0.21), as shown in **Figure 1**.

The univariate Cox regression for the whole study cohort is shown in **Table 2**. On multivariate analysis, ECS (HR: 2.21, 95% CI: 1.21 – 4.04, p = 0.01) and incomplete resection (R-stage; HR: 2.01, 95% CI: 1.10 – 3.69, p = 0.02) remained statistically significant for OS. For RFS, ECS (HR: 1.82, 95% CI: 1.15 – 2.86, p = 0.01), incomplete resection (HR: 1.67, 95% CI: 1.04 – 2.71, p = 0.04) and vascular infiltration of the tumor (V-stage; HR: 2.15, 95% CI: 1.08 – 4.27, p = 0.03) remained statistically significant.

**Table 2:** Univariate Cox regression showing associations between histopathological risk factors and RFS.

	OS			RFS		
	HR	95% CI	p-value	HR	95% CI	p-value
POCRT initiation time	0.98	0.77 – 1.27	0.25	1.10	0.66 – 1.84	0.70
POCRT package time	1.02	0.93 – 1.12	0.71	1.03	0.95 – 1.12	0.50
Age	1.02	0.99 – 1.05	0.14	1.00	0.98 – 1.02	0.63
Gender	1.14	0.66 – 1.96	0.64	1.38	0.93 – 2.10	0.12
Advanced disease stage (pT3-4)	1.10	0.85 – 1.41	0.49	1.10	0.92 – 1.33	0.29
Depth of invasion	0.97	0.85 – 1.10	0.61	0.95	0.87 – 1.04	0.26
Cervical nodal disease (pN+)	1.28	0.65 – 2.54	0.48	1.26	0.77 – 2.08	0.35
Extracapsular spread	2.16	1.19 – 3.93	0.01	1.93	1.25 – 3.00	< 0.01
Margin status	1.03	0.59 – 1.81	0.92	1.39	0.92 – 2.10	0.12
Vascular infiltration	2.35	0.94 – 5.90	0.07	3.10	1.60 – 5.96	< 0.01
Lymphatic infiltration	1.41	0.77 – 2.58	0.27	1.43	0.92 – 2.20	0.11
Incomplete resection	1.96	1.07 – 3.59	0.03	1.71	1.07 – 2.73	0.03
Grade of differentiation	1.16	0.69 – 1.96	0.58	0.98	0.67 – 1.44	0.93

POCRT, postoperative chemoradiation.

## Discussion

Non-adherence to treatment and treatment delays remain a challenging aspect in cancer treatment. Patients who were recommended for AT due to high-risk constellations but refused therapy entirely have a significantly higher risk of recurrence (17). However, the impact of a treatment delay in cases of complete performed AT is still not fully understood. The time gap between surgery and adjuvant treatment or delays in the process of radiation overall may cause microscopic cancer remnants to proliferate and also may promote radioresistance (18). However, tumor biology varies among cancer entities, even in close anatomical subsites such as the oral cavity and the oropharyngeal space (19). For example, HPV-positive oropharyngeal squamous cell carcinoma (OPSCC) has shown increased response rates to radiation therapy and improved survival compared to OSCC (20, 21). Nevertheless, those anatomical subsites are commonly grouped in studies as HNSCC, despite their differences.

There have been several studies investigating the influence of time delays in receiving adjuvant radiation on the oncological outcomes of HNSCC. What all of these studies have in common is that PORT/POCRT was considered in combination, while our cohort only included POCRT. In the study by Graboyes et al. including 41.291 HNSCC patients from the National Cancer Database (NCDB), survival was significantly decreased if the PORT/PORCT initiation time was beyond 6 weeks (adjusted HR: 1.13; 95% CI: 1.08 – 1.19) (11). While an early start of PORT/POCRT had no benefit, increasing durations over 7 weeks were associated with small progressive survival decrements. In addition, Mazul et al. assessed the delay in radiation duration comparing primary and adjuvant therapy in HNSCC patients from the NCDB (12). In their study, radiation duration over 75 days was significantly associated with decreased survival. However, both studies combined several HNSCC entities without stratification for OSCC. In the study by Harris et al., which includes 25.216 HNSCC patients also from the NCDB, multivariate analysis according to the tumor subsite revealed that PORT/POCRT initiation delay was significantly associated with reduced OS for hypopharyngeal SCC, tonsil SCC, and OPSCC, but not OSCC and laryngeal SCC (13). For these tumor subsites, the effect of a delay was neither statistically significant nor clinically meaningful. In the study of Franco et al., 168 HNSCC patients in a single-center setting were investigated, while the PORT/POCRT initiation time was dichotomized at 92 days using the receiver operating characteristic method (22). There was no significant association between initiation time and LRCR, but an overall package time over 150 days was significantly associated with reduced LRCR. It must be noted however that these delay times are considerably over the recommended time limits from the guidelines.

Several other studies have focused on OSCC in particular. Chen et al. investigated the influence of the overall package time in OSCC patients, and reported a significant decrease in OS and RFS when the time was 11 weeks and beyond, which is in accordance with the German national and ESMO guideline recommendations (23). In this study, early staged diseases were also included. In the study by Cheng et al. investigating 8.986 OSCC patients from the Taiwanese national database, PORT/POCRT initiation time beyond 7 weeks only was associated with an adverse trend in survival (24). However, prolonged package time was a significant predictor for worse survival besides ECS and positive surgical margins, which were also outcome predictors in our study. Metzger et al. investigated the influence of the initiation time between admission and surgical intervention on OSCC, reporting a significant influence of delay on the survival for early-staged diseases, but not advanced stages (25). While this aspect of treatment delay was not the subject of our study, the results support the hypothesis of an increased impact of treatment delay on early disease stages.

In our study, we identified a statistically non-significant association between POCRT treatment delay and survival in advanced stage disease. In addition, the differences in survival (OS: 0.2%; LCRC: 4.8%; RFS: 7.1%) were not clinically meaningful. However, these results do not endorse delays in recommended therapy, but might justify a smaller delay in cases of extended surgical therapy due to transplant failure, or a wound healing disorder that may prolong time to initiation. Graboyes et al. investigated the aspect of treatment adherence in HNSCC patients (9). In their study, treatment delay was significantly associated with increased hospital stays and unplanned readmissions within 30 days, suggesting unplanned surgical complications as a cause. In this study, patients with a POCRT initiation delay had significantly longer time durations between surgery and the recommendation of AT from the interdisciplinary tumor board, with a difference of 6.5 days (1 week). The main component of delay was between the recommendation of AT and the start of AT, with a statistically significant difference of 16.5 days (2.4 weeks) between both groups. However, conclusions cannot be drawn regarding whether this delay was patient-related or provider-related.

Our analysis has some limitations. Due to the relatively small sample size as compared to larger database studies, the results must be interpreted with caution. However, larger databases and big data studies have an increased risk of selection bias by assigning patients into two study groups without necessarily stratifying for confounders (26, 27). The aim was therefore to reduce confounding bias by focusing on advanced OSCC and POCRT as the sole AT regime, as well as by considering group equality regarding histopathological risk markers.

## Conclusion

A delay in POCRT initiation up to 11 weeks for advanced OSCC was not a statistically significant risk predictor for survival in this study cohort. As a major part of delay was between the recommendation of AT from the interdisciplinary tumor board and the start of AT, patients may be specifically advised to prevent further delay. In cases of prolonged surgical treatment due to management of complications, a small delay in AT beyond the recommended time limit may be justified and AT should still be pursued. Nevertheless, further evidence is required to determine the influences of specific subgroups and risk constellations.

## Data availability statement

The original contributions presented in the study are included in the article/supplementary material. Further inquiries can be directed to the corresponding author.

## Ethics statement

The studies involving humans were approved by Ethics committee of the Charité Berlin. The studies were conducted in accordance with the local legislation and institutional requirements. Written informed consent for participation was not required from the participants or the participants’ legal guardians/next of kin in accordance with the national legislation and institutional requirements.

## Author contributions

FM: Conceptualization, Data curation, Formal analysis, Funding acquisition, Investigation, Methodology, Project administration, Resources, Software, Supervision, Validation, Visualization, Writing – original draft, Writing – review & editing. MA: Data curation, Writing – review & editing. MR: Writing – review & editing. ES: Data curation, Methodology, Writing – review & editing. CD: Writing – review & editing. KiK: Writing – review & editing. CR: Writing – review & editing. MB: Writing – review & editing. KoK: Writing – review & editing. MH: Writing – review & editing. SK: Formal analysis, Project administration, Supervision, Validation, Writing – review & editing.
